# Using Evidence to Combat Overdiagnosis and Overtreatment: Evaluating Treatments, Tests, and Disease Definitions in the Time of Too Much

**DOI:** 10.1371/journal.pmed.1001655

**Published:** 2014-07-01

**Authors:** Ray Moynihan, David Henry, Karel G. M. Moons

**Affiliations:** 1 Centre for Research in Evidence-Based Practice, Bond University, Robina, Queensland, Australia; 2 University of Toronto, Toronto, Ontario, Canada; 3 Institute for Clinical Evaluative Sciences, Toronto, Ontario, Canada; 4 Julius Center for Health Sciences and Primary Care, University Medical Centre Utrecht, Utrecht, The Netherlands

## Abstract

Ray Moynihan and colleagues outline suggestions for improving the way that medical evidence is produced, analysed, and interpreted to avoid problems of overdiagnosis and overtreatment.

*Please see later in the article for the Editors' Summary*

**Summary Points**

- Overdiagnosis and related overtreatment are increasingly recognised as major problems.
- “Positive” average results from trials of treatments can mask situations where many participants at low risk of disease may receive no benefit.
- The evaluation of diagnostic tests usually involves assessing how well tests detect presence versus absence of a certain disease—rather than how well they detect clinically meaningful stages of disease.
- Changes to disease definitions typically do not involve evaluation of potential harms of overdiagnosis, and are often conducted by heavily conflicted panels.
- We offer suggestions for improving the way evidence is produced, analysed, and interpreted, to help combat overdiagnosis and related overtreatment. These include routine consideration of overdiagnosis and related overtreatment in studies of tests and treatments, and clearer stratification by baseline risk to identify treatment thresholds where benefits are likely to outweigh harms.

While a large part of the world's population faces the problems of underdiagnosis and undertreatment, it is apparent that a “modern epidemic” of overdiagnosis afflicts high-income countries [1], with tangible human and financial costs of the unnecessary management of overdiagnosed diseases [2],[3]. While there is ongoing debate about how to best describe the problem, narrowly defined, overdiagnosis occurs when increasingly sensitive tests identify abnormalities that are indolent, non-progressive, or regressive and that, if left untreated, will not cause symptoms or shorten an individual's life. Such overdiagnosis leads to overtreatment when these “pseudo-diseases” are conventionally managed and treated as if they were real abnormalities; because these findings have a benign prognosis, treatment can only do harm. More broadly defined, overdiagnosis happens when a diagnostic label is applied to people with mild symptoms or at very low risk of future illness, for whom the label and subsequent treatment may do more harm than good [3].

Among the drivers of overdiagnosis are technological developments producing ever more sensitive imaging and biomarker tests, and changing disease and treatment thresholds that medicalize more people [4]. For example, detection of indolent breast lesions is now recognised as an established risk of mammography screening [5]; widened definitions of chronic kidney disease label many asymptomatic seniors as diseased [6]; lowered thresholds increase concerns about overdiagnosis of attention deficit hyperactivity disorder [7]; and more sensitive imaging methods are causing the treatment of large numbers of potentially benign pulmonary emboli [8].

It's important to note there is a complex interrelationship between overdiagnosis and overtreatment—which can occur for many reasons other than overdiagnosis. If we consider the narrow definition of overdiagnosis—where someone is diagnosed with a “disease” that will not progress or harm them—overdiagnosis generally leads to overtreatment. Writing about overdiagnosis in 1998, Black described the cycle of increasingly sensitive tests causing more “pseudo-disease” to be diagnosed and conventionally treated [9]. Because prognosis of “pseudo-disease” is generally benign, there is a perception that patients do well on treatment, reinforcing belief in the value of treatment to the widened patient pool, and in turn fuelling further overtreatment [9]. In other situations, inappropriate overtreatment can occur where there is a legitimate clinical diagnosis, and in some circumstances a degree of overtreatment may be warranted, for instance, the early use of parenteral antibiotics in someone suspected of having bacterial meningitis.

Considering the broader definition of overdiagnosis—involving the medicalization of people with mild problems or at very low risk of disease—it becomes more difficult to define what constitutes subsequent overtreatment. Those judgements will depend on a complex mix of evidence about individual risk, prognosis, and treatment benefit–harm calculations, combined with the personal values and preferences inherent in any decision-making. Cognisant of this complex context, this essay explores how the production, analysis, and interpretation of evidence—whether from individual studies or systematic reviews—might be improved to better inform those judgements, and to better understand and combat the challenges of overdiagnosis and related overtreatment.

## Average Therapeutic Trial Results Can Mislead

It's widely recognised that average treatment effects estimated by systematic reviews of primary therapeutic trials don't really apply to any single patient, and an average benefit can mask both positive and negative effects in different patient subgroups. This leads to treatment of patients who don't benefit, and may suffer harms. Almost two decades ago, advocates of the then emerging evidence-based approach stressed the importance of a nuanced application of evidence from primary trials and systematic reviews for individuals, taking into account a person's absolute risk of an outcome and the need to weigh up potential benefits and harms [10].

More recently Kent and colleagues cited examples where positive clinical trial results masked a lack of meaningful benefit for those at lower risks of illness, including trials involving statins, anticoagulant therapies, and some common surgical procedures [11]. The authors argued that this problem of trials masking the “heterogeneity of treatment effects” can result in guidelines that promote overtreatment, as well as undertreatment, and they recommended estimation of treatment effects after stratifying trial participants according to baseline risk.

Similarly, in a presentation to the inaugural Preventing Overdiagnosis Conference in 2013, Llewelyn re-analysed trial data involving medication for diabetic microalbuminuria and identified subsets of trial participants according to their specific disease stage, finding that many people were likely being treated without benefit [12]. The hope is that better stratification of people by disease stage, or baseline risk of relevant outcomes, will enable better identification of who will benefit and who will be harmed by an intervention, potentially informing the development of more appropriate diagnostic cut-points and treatment thresholds, ultimately reducing overdiagnosis and overtreatment.

## We Need More Nuanced Evaluation of Tests, Too

Just as with the average treatment effects of therapeutics, the average accuracy of a test does not apply to everyone [13]. Moreover, disease is often not simply “present” or “absent”, but rather exists on a continuous scale [14]. Hence, assessing a diagnostic test is more complex than simply knowing its average sensitivity and specificity or how well it detects the presence or absence of a disease [13]. There is a need to know how well diagnostic tests detect subsets of clinically meaningful, as opposed to non-meaningful, abnormalities or disease stages. In other words, it's important to diagnose or identify the spectrum of individuals for whom a disease label and associated intervention will do more good than harm.

A more sophisticated approach is particularly needed when assessing newer, highly sensitive tests—often more costly and burdensome to perform—that can identify earlier, milder, or indolent abnormalities or disease stages. For example, computed tomography pulmonary angiography has led to a dramatic increase in detection of small “sub-segmental” pulmonary emboli, of uncertain clinical significance, with emerging debate over whether many people are being treated unnecessarily with anticoagulants [8]. As a result, pulmonary embolism has been described as a “model for the modern phenomenon of overdiagnosis” [1].

## The Benefits and Harms of Expanding Disease Definitions

A recent investigation of panels that change disease definitions found that while lowering diagnostic thresholds and widening definitions are common, few panels reported on the potential harms of expanding the numbers of people who qualify for a diagnosis [4]. Among panels that had made recent changes to the definitions of common conditions—such as hypertension, attention deficit hyperactivity disorder, and myocardial infarction—the study also found widespread conflicts of interest. For panel publications that included disclosure sections, around 75% of panel members disclosed multiple financial ties to pharmaceutical companies active in the relevant therapeutic area.

Without doubt there are many cases where lower diagnostic thresholds and earlier diagnosis and treatment of disease or risk factors can improve health outcomes. For example, early diagnosis of hypertension helps precipitate preventive lifestyle changes or medication use. However, increasing medicalization may bring harms as well as benefits, as many others have highlighted in debates about “disease mongering” [15]. When, for example, conditions such as restless legs syndrome or female sexual dysfunction are constructed and promoted as being widespread and severe [15], there are legitimate concerns that diagnosing and treating those with mild problems may do them more harm than good.

## Improving the Evidence Base to Combat Overdiagnosis and Overtreatment

As a matter of urgency, the potential for overdiagnosis and related overtreatment should be routinely considered for inclusion in the introduction and discussion sections of reports of studies of therapies, studies of diagnostic test accuracy, systematic reviews of those studies, clinical guidelines, and changes to disease definitions (Box 1). Second, there is a clear need for more research—both original studies and reviews of studies—into the nature and extent of overdiagnosis and related overtreatment within specific conditions—as, for example, has occurred with studies on the risks associated with mammography [5]. Third, the potential harms associated with new treatments and tests, or expanded disease definitions, demand much greater attention in primary studies and reviews.

**Box 1. Summary of Suggestions for Improving the Evidence Base to Combat Overdiagnosis and Related Overtreatment**

1. Routine consideration of overdiagnosis and related overtreatment in the introduction and discussion sections of primary studies and systematic review articles about tests and treatments
2. More condition-specific studies and reviews on the risk of overdiagnosis and related overtreatment—e.g., diagnosis of pulmonary embolism
3. More rigorous routine evaluation of potential harms of treatments, tests, and changes to disease definitions
4. In studies and reviews of studies of therapies, clearer stratification by baseline risk, to better identify treatment thresholds where benefits are likely to outweigh harms
5. In studies and reviews of studies of test accuracy, more clarity about which target condition or spectrum of a disease is being considered, with a shift from a dichotomous “disease/no disease” frame to a “spectrum of disease severity” frame, and a linking of test accuracy to consequences for treatment and patient outcomes
6. Panels that review and change disease definitions that are free of conflicts, and routinely consider evidence for potential harms as well as potential benefits of the changes they propose

For evaluation of treatments, more clarity is required about the specific definitions of diseases being treated in primary treatment studies and subsequent systematic reviews. As per the recommendations of Kent and colleagues [11], clearer stratification of groups at varying degrees of baseline risk or disease stage is needed, to better identify treatment thresholds at which the harms of treatment start to outweigh benefits. Sometimes this will require re-analysis of large (e.g., pooled individual participant) datasets, underscoring the need for access to raw data from trials.

For primary studies and reviews of studies of diagnostic test accuracy, there is a need to make explicit exactly which stages or spectrum of a target disease is being considered—also referred to as the “target condition” [14]. Where possible, it may be desirable to shift the paradigm from a dichotomous frame—disease presence versus absence—to thinking about a spectrum of disease severity. Moreover, when diagnostic studies show improved detection (or exclusion) of specific disease stages, researchers should try to link the consequences of such improved diagnostic accuracy to subsequent treatment decisions. Ideally, the consequences of such changed treatment decisions for patient outcomes might also be addressed [16]. Such elaborations to conventional diagnostic test accuracy studies would help identify at what diagnostic disease spectrum thresholds subsequent treatments will do more good than harm.

And, finally, the need to improve the process of disease definition—with awareness of the dangers of overdiagnosis and overtreatment—is being increasingly accepted, with international organisations, including the Guidelines International Network, currently looking to develop new guidance. While a detailed debate will ensue in coming years, we believe several key principles might underpin the reform of how disease definitions are changed: panel members should be free of financial and reputational conflicts of interest; strong evidence, ideally from randomised trial data, should demonstrate that the use of new criteria will meaningfully reduce mortality and/or morbidity; and potential benefits and potential harms of labelling and treatment using the new criteria should be explicitly investigated and reported.

## Conclusions

We offer these suggestions as part of the wider scientific debate underway on how to safely and fairly wind back the harms of too much medicine [17]. We are hopeful that a heightened attention to the dangers of overdiagnosis and related overtreatment may lead to an enhanced evidence base on these topics. This, in turn, will help produce fairer, more rational, and less wasteful health care systems, built on a reformed process of disease definition that offers diagnostic labels and medical interventions only to those likely to benefit from them.
